# Supplementary material for: Accommodating Site Variation In Neuroimaging Data Using Normative And Hierarchical Bayesian Models
Source: Neuroimage. Author manuscript; Available in PMC 2023 Jul 17. (PMC7614761; doi:10.1101/2021.02.09.430363)
Supplement: Supplementary Materials [file EMS178735-supplement-Supplementary_Materials.pdf]

Supplementary material

0.1 Missing values and sample sizes per model

Despite the aim of the study to run all seven models in this study on the same data set, we were faced with the challenge that differing requirements for each model with respect to missing values made an adjustment of sample sizes per model necessary. Combat [**Fortin2017**, **Fortin2018**] accepts missing values, and could be thus run on the full data set. In contrast, ComBat Gam [**pomponio2020**] does not. Thus, for ComBat Gam all subjects with a missing value in any of the 35 regions had to be excluded, which lead to a sample size reduction from 391 to 370 individuals for the training set, and from 168 to 156 individuals for the healthy test set. The normative modeling process is performed region wise and independently, thus only the subjects that contained missing subjects for that particular region were deleted.

0.2 Model convergence, effective sample size and $\hat{R}$

For the present project, each model run entailed a Monte-Carlo sampling process of 4000 iterations in Stan, of which 2000 were disregarded as warm up. Stan allows for the computation of a number of diagnostics on the quality of the Markov Chain Monte Carlo (MCMC) sampling process, which are reported in the following.

0.3 Model convergence

Markov chains are defined to only generate samples from the target distribution after the distribution has converged to an equilibrium, thus, when the distribution is considered to be the target density. In theory, this equilibrium can only asymptotically be reached, as the number of draws is theoretically infinite.

In practice, the number of draws has to be a-priori set to a finite amount. As a consequence, the actual and convergence to the target density has to be monitored [**carpenter2017stan**, **Gelman2015**, **stan2019**]. One way to monitor the convergence of a chain to equilibrium is to compare the chain to other randomly initialized chains. This can either be done via visual inspection, or using the scale reduction statistic, $\hat{R}$ [**gelman1992inference**]. For visual inspection, the trace plots over 4 chains for all parameters can be found in Figs. 1a, 2a , 3a, 4a, 5a, 6a, 7a. The $\hat{R}$ values, indicating the convergence of chains, can be found in Figs. 1b, 2b , 3b, 4b, 5b, 6b, 7b for each model, respectively. All $\hat{R}$ values are $<$1.05, which provides good evidence that all chains have reached convergence and can therefore be considered to provide unbiased samples from the target density.

0.4 Statistical comparison of measures of model performance

All comparisons regarding measures of model performance were performed using two-way ANOVAs including the factors model (HBLM, HBGPM, Combat Gam, ComBat, ComBat without covariates, residuals, raw data) and set (train, test). Post hoc tests were performed using Tukey tests and corrected for multiple comparisons. Parametric tests such as ANOVA were deliberately chosen over their non-parametric equivalents, since deviations from gaussianity were negligible in the present data set and in the authors' opinion, the substantial loss of power with the choice of non-parametric tests does not scale with the potential threat of violated modeling assumptions such as homoscedasticity and gaussianity.

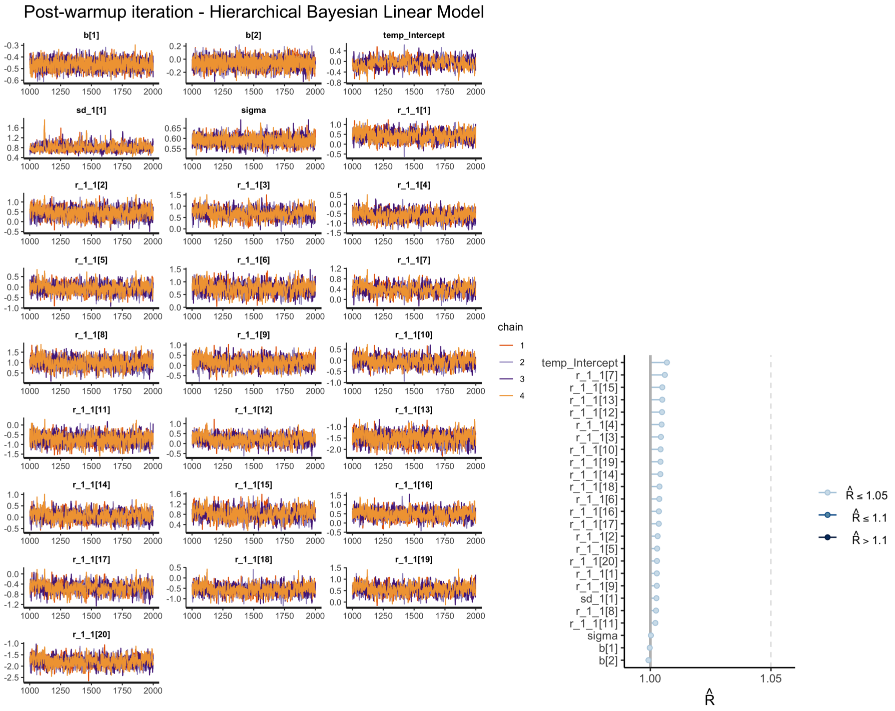

(a) Trace plots

(b) $\hat{R}$

Figure 1: Hierarchical Bayesian Linear Model

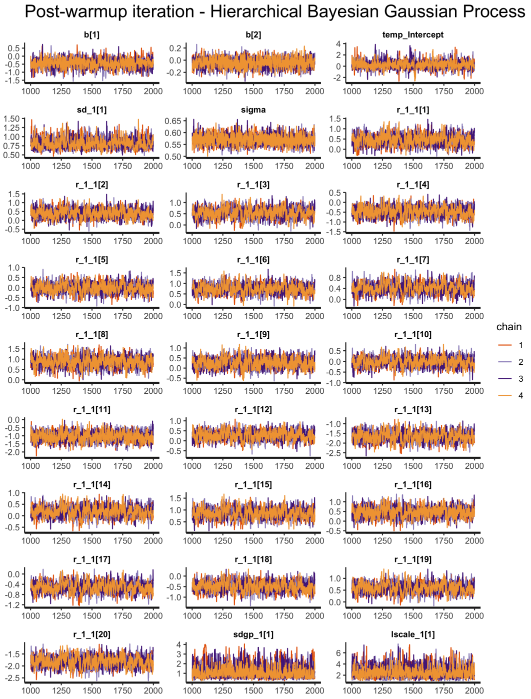

(a) Trace plots

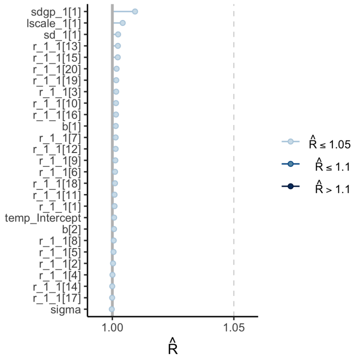

(b) $\hat{R}$

Figure 2: Hierarchical Bayesian Gaussian Process Model

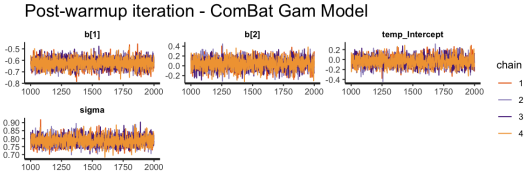

(a) Trace plots

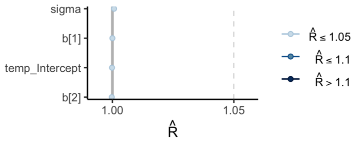

(b) $\hat{R}$

Figure 3: ComBat Gam Model

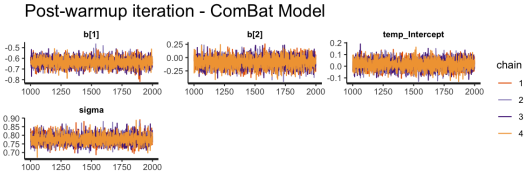

(a) Trace plots

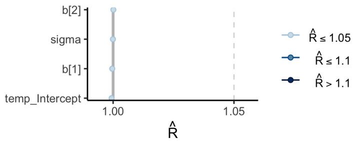

(b) $\hat{R}$

Figure 4: ComBat Model

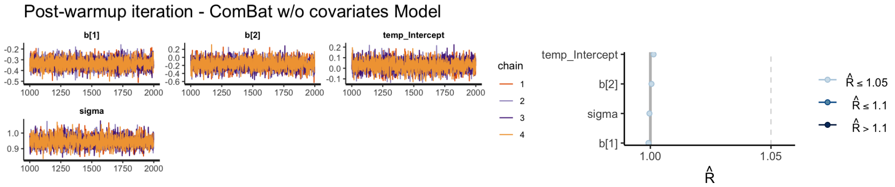

(a) Trace plots

(b) $\hat{R}$

Figure 5: ComBat w/o covariates Model

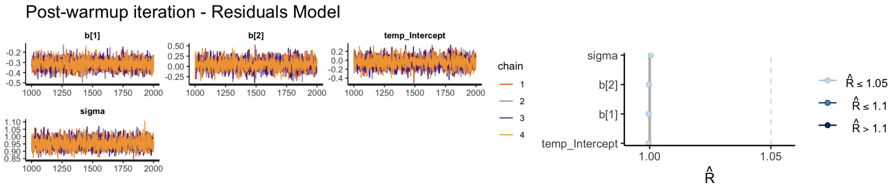

(a) Trace plots

(b) $\hat{R}$

Figure 6: Residuals Model

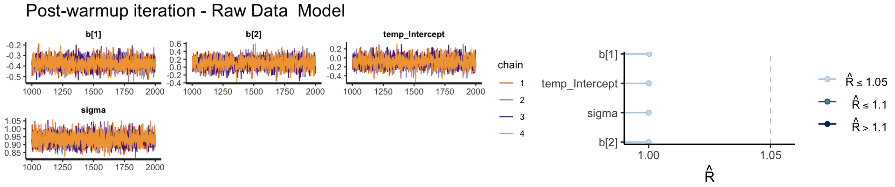

(a) Trace plots

(b) $\hat{R}$

Figure 7: Raw Data Model

0.4.1 Effective sample size

One characteristic of MCMC methods is that samples will be auto- or anti-correlated within a chain, leading to a reduction of precision in the estimates of posterior quantities [**geyer2011introduction**]. Stan uses the auto correlation ρ_t between samples n and $n{+}t$ with lag t to estimate the effective samples size N_{eff} of independent samples in the chain. N_{eff} is considered to have the same estimation power as N correlated samples and is then used, rather than N, to estimate precision and error measures [**stan2019**]. N_{eff} for all models can be found in Figs. 8a, 8b, 8c, 8d, 8e, 8f, 8g.

0.5 Results

0.6 Correlation between true and predicted value

Correlations between true and predicted values for the HBLM and HBGPM are expressed in terms of the correlation coefficient ρ, calculated separately for each region. ρ ranged from 0.60 to 0.84 in the training and 0.55 to 0.80 in the test set. Overall, for just the Bayesian models, correlations were higher in the training set and dropped in the test set (training set, across all regions: $\bar{\rho}_{HBLM} = 0.73$, $SE = 0.05$; $\bar{\rho}_{HBGPM} = 0.75$, $SE = 0.06$; test set: $\bar{\rho}_{HBLM} = 0.69$, $SE = 0.06$; $\bar{\rho}_{HBGPM} = 0.69$, $SE = 0.06$, $F[1, 136] = 18.82$, $p < 0.0001$). Correlations did not differ significantly between the HBLM and HBGPM. ($F[1, 136] = 2.16$, $p = 0.14$).

Comparisons to the other, non-Bayesian models showed that ρ was significantly higher for our models that included *site* as a predictor for with all other models in which site was harmonized for prior to running the normative models, both for training and test set (t-test *HBLM* and any other model $p < 0.001$, t-test *HBGPM* and any other model $p < 0.001$, both for training and test set.) The full distribution of the correlation coefficient ρ for all 35 regions per model

can be found in Fig 3a, main text. In addition, a test comparing the performance of all models for all regions (Bayesian and non-Bayesian) showed that predictions made from training data were not overall more accurate than predictions from the test data (main effect *set*, F[1, 476] = 0.30, p = ns, interaction *set* × *model*, F[1, 476] = 3.50, p = 0.002). Further inspection showed that this might have been caused by the *residuals*, the *ComBat w/o covariates* and the *ComBat* model, where the test set performed *better* than the training set, canceling out performance benefits of the training data in the HBLM and HBGPM (see also Fig. 3a, main text).

0.7 Standardized Root Mean Squared Errors

We further evaluated the fit of the models by calculating the Standardized Root Mean Squared Error (SRMSE) between true values and predicted values per model per region. As expected, the SRMSE was larger for the test set (M = 0.083) and smaller for the training set (M = 0.080, [F(1, 134278) = 59.28, p $<$ 0.001]). For both the training and the test set, the Bayesian models showed smaller SRMSEs than all other models across all regions (p $<$ 0.001; training set: $SR\bar{M}SE_{HBGPM} = 0.06$, $SE = 0.005$; $SR\bar{M}SE_{HBLM} = 0.06$, $SE = 0.005$; $SR\bar{M}SE_{ComBatGam} = 0.11$, $SE = 0.01$; $SR\bar{M}SE_{residuals} = 0.09$, $SE = 0.002$; $SR\bar{M}SE_{ComBat} = 0.08$, $SE = 0.007$, $SR\bar{M}SE_{ComBat-w/o-covariates} = 0.09$, $SE = 0.002$; $SR\bar{M}SE_{rawdata} = 0.08$, $SE = 0.005$; test set: $SR\bar{M}SE_{HBGPM} = 0.06$, $SE = 0.005$; $SR\bar{M}SE_{HBLM} = 0.07$, $SE = 0.006$; $SR\bar{M}SE_{ComBatGam} = 0.12$, $SE = 0.01$; $SR\bar{M}SE_{residuals} = 0.09$, $SE = 0.005$; $SR\bar{M}SE_{ComBat} = 0.08$, $SE = 0.009$; $SR\bar{M}SE_{ComBat-w/o-covariates} = 0.09$, $SE = 0.005$; $SR\bar{M}SE_{rawdata} = 0.085$, $SE = 0.007$). Neither in the training nor the test set did the Bayesian models differ from each other (training set: contrast *HBLMR* - *HBLM*, t = 2.33, p = *ns.*; test set: contrast *HBLMR* - *HBLM*, t = 1.14, p = *ns.*). We also

observed that both in the training and test set, the SRMSE of *ComBat w/o covariates* did not differ from the SRMSE of the *residuals* (training set: contrast *ComBat w/o covariates - residuals*, t = 0.69, p = *ns.*; test set: contrast *ComBat w/o covariates - residuals*, t = -1.70, p = *ns.*. The full distribution of SRMSE for all 35 regions per model can be found in Fig 3b, main text.

0.8 Explained variance

Analysis of the proportion of variance explained EV = $\frac{\sigma^2_{\hat{y}-y}}{\sigma^2_y}$ per model per region were in line with the results reflected in ρ and SRMSE. EV was higher for the *HBLM* and *HBGPM*, with an average of 0.56 (*HBGPM*, range: 0.35-0.70) and 0.53 (*HBLM*, range 0.35 - 0.67) for the training set and 0.50 (*HBGPM*, range 0.31 - 0.63) and 0.48 (*HBLM*, range 0.28 - 0.60) for the test set across all cortical regions. The proportion of explained variance was substantially lower for the comparison models, with the ComBat and the ComBat Gam model performing best out of the comparison models, with an average of 0.31 for ComBat model for the training set (range: 0.00 - 0.51) and 0.33 for the test set (range -0.02 - 0.58) across cortical regions, and an average of 0.31 for ComBat Gam for the training set (range: -0.01 - 0.51) and 0.22 for the test set (range 0.03 - 0.46) but showing lower EV than the Bayesian models. Predictions derived from *residuals* and *ComBat w/o covariates* showed even lower EV, with *residuals* explaining an average of 0.07 for the training set (range: 0.00 - 0.15) and 0.11 for the test set (range 0.00 - 0.20) across cortical regions, and *ComBat* explaining an average of 0.09 for the training set (range: 0.00 - 0.17) and 0.12 for the test set (range: 0.00 - 0.25) across cortical regions. Thus, the *ComBat w/o covariates, ComBat and residuals* model performed even worse than predictions derived from *raw data*, which showed an average EV of 0.21 in the training set (range: 0.00 -0.46) and 0.20 in the test set (range 0.00 - 0.44) across cortical regions. These results

include the interesting finding that the test set shows slightly higher EVs than the training set for all comparison models. An overview over the distribution of explained variance for training and the test set for all 35 regions for all models can be found in Fig. 3c, main text.

0.9 Log likelihood

The point-wise log likelihoods (LL) between the true and predicted were calculated for each data point, summed up per model across regions and averaged y the number of individuals in training and test set per model, respectively. The averaged summed LL across regions was closer to zero for the nonlinear Bayesian model than for the linear Bayesian model, both for the training and the test set ($\sum \frac{1}{n_{test}} LL_{HBGPM}$, test set: -1.109, $\sum \frac{1}{n_{test}} LL_{HBLM}$ test set: -1.121; $\sum \frac{1}{n_{train}} LL_{HBGPM}$, training set: -1.020, $\sum \frac{1}{n_{train}} LL_{HBLM}$, training set: -1.05. LL values were less close to zero for all comparison models, with the *Combat* model performing best for among those models, followed by the *raw data* model, the *residuals* model and the *ComBat w/o covariates* model (an overview of the log likelihood for all models is given in Tab. 4, main text) The distribution of the log likelihood for all regions is given in Fig. 3d, main text.

0.10 Effect sizes for site: Raw data and after correction with models

Effect sizes in from of in form of partial η^2 and corresponding p values for raw data and after correction with models. Please see also a commentary on the use of effect sizes for site effect correction in the main text.

**Raw data**	**partial η^2**	**p for site**
Training set	0.58	<0.0001
Test set	0.55	<0.0001
Autism test	0.51	<0.0001

Table 1: Effect sizes for site, raw data.

**Test set (controls)**	**partial η^2**	**p for site**
HBGPM	0.08	0.03
HBLM	0.17	0.06
ComBat Gam	0.15	0.2
Combat	0.04	0.99
Combat w/o Sex & Site	0.05	0.98
Residuals	0.04	0.99

Table 2: Effect sizes for site after correction with various models, control test set.

**Training set (controls)**	**partial η^2**	**p for site**
HBGPM	<0.001	1
HBLM	<0.001	1
ComBat Gam	0.03	0.96
Combat	0.01	0.99
Combat w/o Sex & Site	0.06	0.2
Residuals	0.06	0.3

Table 3: Effect sizes for site after correction with various models, control training set.

**Autism test set**	textbfpartial η^2	**p for site**
HBGPM	0.19	0.04
HBLM	0.08	0.01

Table 4: Effect sizes for site after correction with HBLM and HBGPM, autism test set.

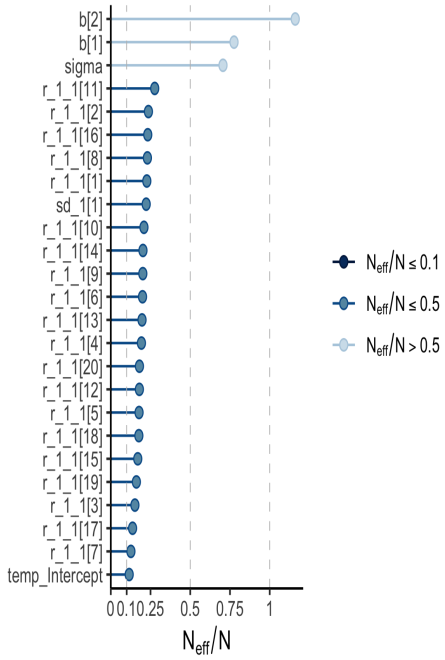

(a) Hierarchical Bayesian Linear Model

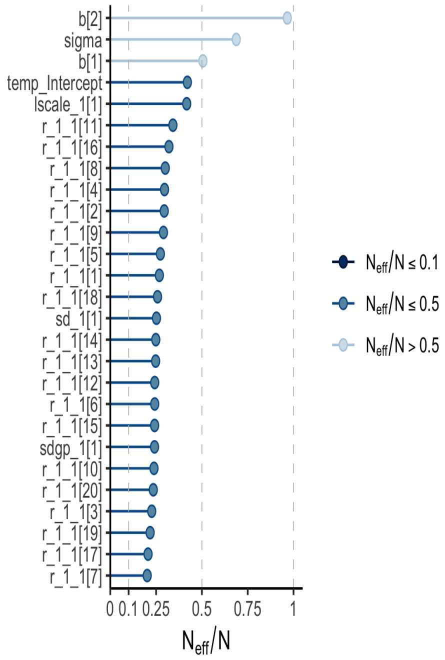

(b) Hierarchical Bayesian Gaussian Process Model

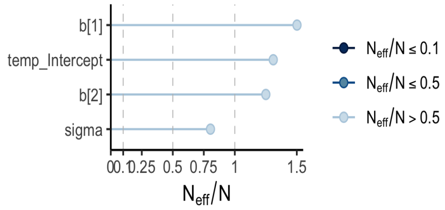

(c) Model ComBat w/o covariates.

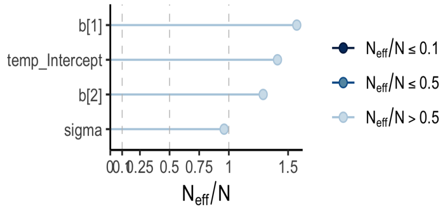

(d) Model ComBat w age/sex preserved

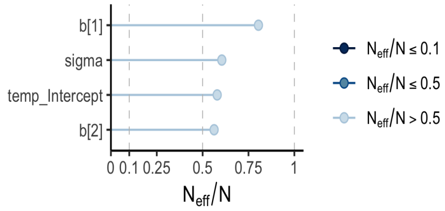

(e) Residuals Model

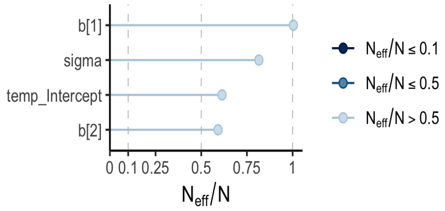

(f) Raw data Model

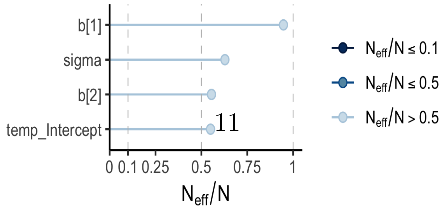

(g) ComBat Gam Model

Figure 8: Effective sample sizes N_{eff} for all parameters
